# Potential Crosstalk Between Parkinson's Disease and Energy Metabolism

**DOI:** 10.14336/AD.2021.0422

**Published:** 2021-12-01

**Authors:** Meiqiu Liu, Qian Jiao, Xixun Du, Mingxia Bi, Xi Chen, Hong Jiang

**Affiliations:** Department of Physiology, Shandong Provincial Key Laboratory of Pathogenesis and Prevention of Neurological Disorders and State Key Disciplines: Physiology, School of Basic Medicine, Medical College, Qingdao University, Qingdao, China

**Keywords:** Parkinson’s disease, Energy metabolism, Metabolism-related peptides, Obesity, T2DM, Hypercholesterolemia

## Abstract

Parkinson's disease (PD) is characterized by the accumulation of alpha-synuclein (α-Syn) in the substantia nigra (SN) and the degeneration of nigrostriatal dopaminergic (DAergic) neurons. Some studies have reported that the pathology of PD originates from the gastrointestinal (GI) tract, which also serves as an energy portal, and develops upward along the neural pathway to the central nervous system (CNS), including the dorsal motor nucleus of vagus (DMV), SN, and hypothalamus, which are also involved in energy metabolism control. Therefore, we discuss the alterations of nuclei that regulate energy metabolism in the development of PD. In addition, due to their anti-inflammatory, antiapoptotic and antioxidative roles, metabolism-related peptides are involved in the progression of PD. Furthermore, abnormal glucose and lipid metabolism are common in PD patients and exacerbate the pathological changes in PD. Therefore, in this review, we attempt to explain the correlation between PD and energy metabolism, which may provide possible strategies for PD treatment.

## 1. Introduction

**Figure 1.**
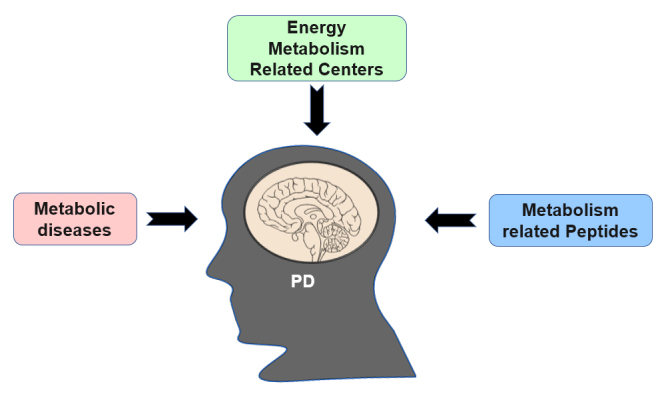
**Relationship between PD and energy metabolism.** The relationship is discussed from three aspects in this review: energy metabolism-related centers, metabolism-related peptides and metabolic diseases.

Parkinson's disease (PD) is the second most prevalent neurodegenerative disease and characterized by tremors, rigidity, dystonia, bradykinesia, and postural instability. The typical pathological manifestations of PD are the progressive loss of dopaminergic (DAergic) neurons and the formation of Lewy bodies (LBs) in the substantia nigra (SN). Risk factors for PD include age, genetic susceptibility, the environment, oxidative stress, mitochondrial dysfunction, inflammation, and energy imbalance [1, 2]. Some studies show that obesity increases the risk of PD, but the body weight of most patients with PD is lower than those of healthy people and partially recover after effective treatments, such as the application of dopamine (DA) agonists and deep brain stimulation (DBS) surgery [3]. LBs also appear in other central nuclei involved in the regulation of energy metabolism in patients with PD. In addition, a few metabolism-related peptides affect the pathological development of PD; for example, they can increase DAergic neuron firing, reduce oxidative stress, and improve motor symptoms and cognitive functions. Additionally, some PD patients and people with metabolic diseases have overlapping symptoms, such as insulin resistance. Recent studies even indicate that energy metabolism is a potential target for PD prevention and treatment [4]. All of the above results indicate the close correlation between PD and energy metabolism. Therefore, in this manuscript, we further highlight recent evidence and discuss the correlation between PD and energy metabolism (Fig. 1).

## 2. Parkinson's disease and energy metabolism-related centers

**Figure 2.**
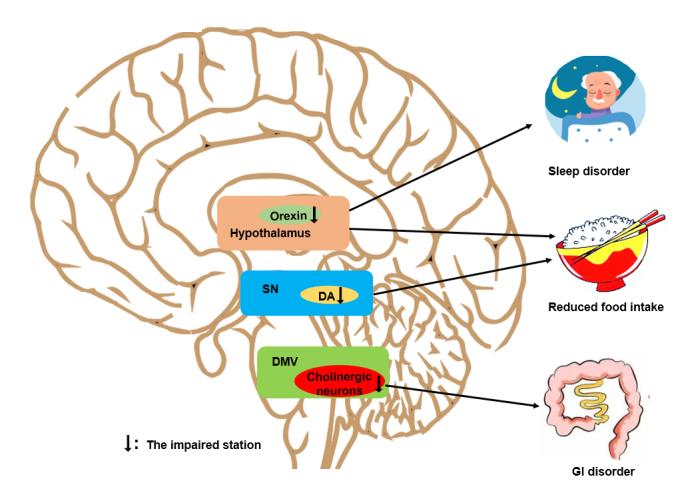
**The major impacts of alterations in energy metabolism-related centers on PD.** In the DMV, the impairment of cholinergic neurons is involved in the inhibition of GI motility in patients with PD. In the SN, the loss of DAergic neurons affects food intake. In the hypothalamus, abnormal orexinergic neurons induce a reduction in food intake and sleep disorders.

Some studies postulate that the pathogen of PD originates from the enteric nervous system (ENS) and spreads to the central nervous system (CNS) via the vagus nerve, in turn passing through the dorsal motor nucleus of vagus (DMV) and the SN, which are involved in energy metabolism regulation [5-7] Kim et al. confirmed that the phosphorylation of serine 129 of alpha-synuclein (pSer129-α-Syn) was detected in multiple central nuclei, including the DMV and SN, after the injection of pathological α-Syn fibrils into the duodenal and pyloric muscularis layers [8]. Additionally, in PD patients, α-Syn aggregation is observable in the hypothalamus, a classic area of energy homeostasis control [9]. Because these nuclei are involved in the regulation of energy metabolism, we herein discuss the relationships of PD with the DMV, SN and hypothalamus (Fig. 2).

### 2.1 Dorsal motor nucleus of vagus

Around 70% of patients with PD exhibit gastrointestinal (GI) dysfunctions, especially constipation, gastroparesis, and abdominal distension, which are potentially induced by DMV impairment [10]. The DMV is located in the ventral bulbous medullae, wherein parasympathetic vagus nerve fibers are emitted to innervate the ENS and regulate the secretion, motility and absorption of the GI tract, which is the gateway to energy intake. In the early stage of PD progression, α-Syn accumulates in the DMV [11]. LBs, in which α-Syn is the main component, are reportedly highly present in the axons of DMV neurons, as shown by immunostaining, and the main DMV neurons are cholinergic [12-14]. Many cholinergic neurons, which are vital for regulating gastric emptying in the gastric inhibitory/excitatory vagal motor circuit, tend to be lost in the DMVs of PD patients, and patients with longer disease durations show more serious neuron loss [10, 15].

A series of PD animal models also demonstrated that DMV damage induced GI dysfunction. 6-Hydroxy-dopamine (6-OHDA), lipopolysaccharides (LPS) and rotenone are used to produce PD models, all of which show GI symptoms related to DMV. In 6-OHDA PD models, the tyrosine hydroxylase (TH) immunoreactivity in DMV neurons is significantly increased; however, the expression of choline acetyltransferase (ChAT, a marker of cholinergic neurons) is decreased, the Ach content is reduced in the gastric muscular layer, and gastric emptying is delayed [14]. The administration of LPS into the rat SN results in ChAT alterations in the DMV that are consistent with those of the 6-OHDA models, to impair the gastric motility [16]. Rotenone is shown to inhibit the activity of mitochondrial complex I across the blood-brain barrier, leading to the death of DAergic neurons in the SN [17]. In mice that are intragastrically administered rotenone, α-Syn aggregation is observed in ENS ganglia and ChAT-positive neurons of the DMV and in TH-positive neurons of the SN, and the mice showed GI dysfunctions and PD-like motor symptoms [18].

### 2.2 Substantia nigra

The progressive loss of DAergic neurons in the SN is the major feature of PD. In the midbrain, the SN is located adjacent to the ventral tegmental area (VTA), and both contain abundant DAergic neurons. The SN and VTA project to the dorsal and ventral striataum (Str), respectively, through the medial forebrain bundle (MFB). The DAergic projection from the VTA to the ventral Str plays a vital role in the hedonic control of food intake, and the adjacent SN DAergic neurons are also involved in food intake regulation. A study revealed that food intake enhanced the DA release in the dorsal Str, which was concerned with pleasure of feeding. Several studies demonstrated that the depletion of DAergic neurons in the SN of 6-OHDA rats induced impaired feeding and drinking behaviors, and the body weight of animals with unilateral SN lesions was reduced [19]. The restoration of TH in the dorsal Str of mice lacking TH increased their food intake [20]. In addition, DAergic neurons in the SN participate in the control of digestive movement. Haloperidol induces the death of TH^+^ neurons in the SN pars compacta (SNpc), which significantly weakens vacuous chewing movements (VCMs) in rats [21]. The SN is also related to the regulation of heat production. In 6-OHDA-induced PD models, thermogenic interscapular brown adipose tissue (IBAT) is activated to enhance the decomposition of triglycerides and increase heat production [22].

### 2.3 Hypothalamus

The hypothalamus is the classic center of energy homeostasis regulation, which is also related to the control of body temperature and biological rhythms. Although the hypothalamus is not the main damaged area in patients with PD, hypothalamic LBs have been discovered in these patients [9]. Thermoregulation impairments, such as hypothermia and hyperhidrosis, in PD patients are associated with hypothalamic lesions, which might result from the accumulation of α-Syn in the hypothalamus [23]. Approximately 64% of PD patients also have sleep disorders, which can induce energy imbalance [24]. Sleep disorders in PD patients are related to orexinergic neuronal damage in the hypothalamus, which is associated with both hunger and wakefulness. In fact, orexin system dysfunction plays a role in both daily drowsiness and eating disorders [25]. Low orexin levels are related to the occurrence of narcolepsy in PD patients [26]. In transgenic PD models (*A53T* mice), α-Syn accumulations in hypothalamic orexin neurons were observed in the early stage, and orexin neuronal loss occurred in the late stage, which was accompanied by reduction in fat mass and an increase in energy expenditure [27].

In addition, PD susceptibility genes affect energy metabolism in the hypothalamus. *LRRK2* is a molecule related to the inheritance of PD. Under acute restraint stress, *LRRK2*-knockout (KO) mice exhibit a decreased number of c-Fos-positive cells and alleviated destruction of the extracellular regulated protein kinase (ERK) signaling pathway in the paraventricular nucleus (PVN), as well as improvements in GI dysmotility, which implies that *LRRK2* might be associated with GI dysmotility in the PVN stress pathway [28]. DJ-1 is a protein encoded by the Parkinson disease protein 7 (*PARK7*) gene that inhibits the aggregation of α-Syn via its chaperone activity, and acidic DJ-1 isoforms are considered biomarker candidates for PD [29]. The formation of acidic DJ-1 isoforms is increased in the hypothalamus of mice fed a high-fat diet, which increases the prevalence of PD [30]. *Thy1-aSYN* transgenic mice are a kind of PD model that overexpress the full-length human wild-type (WT) α-Syn protein. *Thy1-aSYN* mice manifest adiposity loss, feeding behavior alterations, and energy expenditure reductions at 6 months of age, which might be related to the reduction in the phosphorylated active form of signal transducer and activator of transcription 3 (STAT3) and mTOR complex 2 (mTORC2) signaling in the hypothalamus [31].

## 3. Parkinson's disease and metabolism-related peptides

**Figure 3.**
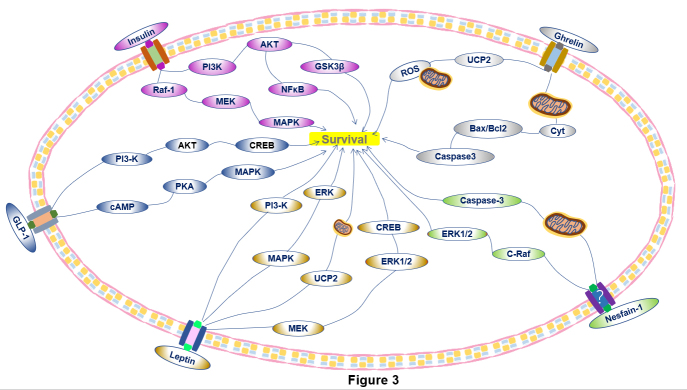
**The major intracellular pathways underlying the protective effect of metabolism-related peptides on DAergic neurons.** Insulin: Activation of the Raf1-MEK-MAPK pathway is involved in the promotion of protein expression to stimulate the growth and maintenance of neurons and synaptic plasticity. The PI3-K-Akt pathway plays a role in apoptosis inhibition and cellular activation as well as in the expression of inflammatory mediators. GSK3β and NFκB are downstream mediators related to the regulation of inflammation in DAergic cells. GLP-1: GLP-1 upregulates intracellular cAMP expression and activates PI3-K, which are involved in two main pathways: cAMP-PKA-MAPK and PI3-K-AKT-CREB. Leptin: Leptin activates the PI3-K and MAPK-ERK pathways, which reduces apoptosis and enhances the survival of DAergic neurons. Leptin also activates the MEK-ERK1/2-CREB pathway to protect cells. Leptin increases UCP2 levels to maintain mitochondrial functions. Ghrelin: Ghrelin exerts its antioxidative effect by reversing Cyt release, increasing the Bax/Bcl-2 ratio and reducing caspase-3 activation. Ghrelin also upregulates UCP-2 and enhances neuroprotection by buffering ROS production. Nesfatin-1: Nesfatin-1, whose receptor remains unknown, decreases the activity of caspase-3 and activates the C-Raf-ERK1/2 pathway to protect DAergic neurons.

Metabolism-related peptides produced in the periphery or the CNS affect energy balance through regulating appetite, GI dynamics and sleep. Research discovered that metabolism-related peptides had neuroprotective effects in PD. Metabolism-related peptides not only regulate energy metabolism but also affect the pathological process of PD by regulating oxidative stress, apoptosis, synaptic plasticity, etc. (Table 1, Fig. 3).

**Table 1:** The main mechanisms and effects of different metabolism-related peptides on PD.

Metabolism-related Peptides	The levels of peptides in PD	Regulatory mechanisms of metabolism related peptide in PD	The effects of the peptides in PD energy variation
Insulin	↓	Activating the Raf1-MEK-MAPK pathway involved in the growth and maintenance of neurons and synaptic plasticity. Inhibiting apoptosis and inflammation through the PI3-K-Akt pathway. Regulating the downstream mediators of GSK3β and NFκB to participate in the regulation of inflammation in DAergic cells.	Decreasing food intake and enhancing glycogen synthesis in peripheral tissue
GLP-1	↓	Upregulating the cAMP- PKA-MAPK and the PI3-K-AKT-CREB pathways to protect the DAergic neurons. Diminishing oxidative stress levels, anti-inflammation via AMP-PGC-1α pathway and decreasing the activation of microglia.	Decreasing food intake.
Leptin	↓	Activating the PI3-K and MAPK-ERK pathways to reduce apoptosis and enhance the survival of DAergic neurons. Activating the MEK-ERK1/2-CREB pathway to protect cell. Increasing UCP2 levels to maintain mitochondrial functions.	Inhibiting food intake.
Ghrelin	↓	Reducing the levels of oxidative stress and apoptosis, upregulating UCP2-dependent mitochondrial mechanisms. Reversing the Cyt release, increasing Bax/Bcl-2 ratio and reducing caspase-3 activation to play antioxidative functions.	Increasing food intake and body weight, affecting the RBD behavior.
Nesfatin-1	↓	Performing anti-inflammation and anti-oxidation via C-Raf-ERK1/2 pathway.	Suppressing food intake.

### 3.1 Insulin

Insulin is a protein hormone secreted by islet β cells stimulated by blood glucose. Insulin promotes the conversion of glucose to glycogen in liver and muscle cells. Type 2 diabetes mellitus (T2DM) is characterized by hyperglycemia, insulin resistance, and a relative lack of insulin that mainly occurs in obese subjects and is very common in middle-aged and elderly people. Studies have shown that the incidence of impaired glucose tolerance in PD patients could be as high as 50%-80% [32]. Prospective studies in Finland and the United States found that the risk of PD in T2DM patients was increased by 85% and 40%, respectively [32]. A subanalysis showed a potential positive correlation between PD and patients diagnosed with T2DM for more than 10 years [33]. Case-control studies in China, Taiwan, and Denmark support that T2DM is a risk factor for the development of PD. Additionally, long-term DA agonist treatment or surgery in patients with PD results in excessive insulin secretion, which is considered to be a factor underlying body mass index (BMI) changes [34].

Impaired glucose tolerance and hyperglycemia are common manifestations in most patients with PD and might be related to DA loss. In a hypoglycemic state, blood glucose levels are controlled by DAergic neurons via the binding of the insulin receptor (IR) in the SN, and the SNpc expression of IR mRNA is significantly downregulated in patients with PD [35].

Insulin binds with IR and activates the phosphorylation of IR substrate (IRS) to trigger secondary messenger pathways [36]. High levels of IRS phosphorylation at serine residues induce the inactivation of insulin signaling in the basal ganglia and SN over time, which might promote DAergic neuronal death [37]. In addition, the mRNA expression of IRS is decreased in PD patients, which accelerates the loss of DAergic neurons [38]. Raf-*1/methyl ethyl ketone* (MEK)-mitogen-associated protein kinase (MAPK)/ERK and phosphatidylinositol-3-kinase (PI3-K)/protein kinase B (AKT) are the main pathways responsible for insulin signaling, and glycogen synthase kinase 3 beta (GSK-3β) is a downstream product of the PI3-K-Akt insulin signaling pathway. Insulin effectually upregulates the phosphorylation of GSK3β at Ser9 to inhibit GSK3β by enhancing the PI3-K/Akt pathway, which most likely ameliorates cognitive disorders in patients with PD [39]. The loss of DAergic neurons is relieved and motor symptoms are improved in 6-OHDA-treated rats administered insulin via the intranasal route [40]. In PD worm models, gastrodin diminishes the accumulation of α-Syn and DAergic neuronal damage through the insulin-like pathway [41].

Insulin resistance is aggravated in PD patients compared with age-matched controls [42]. Recent research suggests that insulin resistance decreases the expression of DA transporters in the SNpc and Str [43]. Although α-Syn aggregation can be prevented by insulin-degrading enzyme (IDE), IDE is competitively restricted by insulin resistance, which promotes α-Syn accumulation in the pancreas [44]. In patients with T2DM, insulin resistance induces the emergence of advanced glycation end-products (AGEs) which interact with their receptors to activate downstream pathways, resulting in oxidative stress, inflammatory reactions, and neuronal death [45]. AGEs were found in the SNs of T2DM patients, and AGEs and α-Syn were found in the LBs of PD patients [45, 46]. Glycation also promotes the aggregation of α-Syn by inducing crosslinks and the formation of α-Syn and/or inhibiting the α-Syn degradation mediated by ubiquitins, proteasomes and lysosomes [45, 46]. While the initial short-term activation of microglia has anti-inflammatory effects, long-term activation aggravates inflammation in patients with PD, and insulin resistance prolongs the microglial activation time and increases the expression of inflammatory mediators via the NFκB and PI3-K/Akt pathways [47, 48]. Relieving insulin resistance was shown to alleviate the age-related anxiety and depressive behaviors induced by increased DA turnover in the Str and mesolimbic system of mice with brain/neuron-specific insulin receptor knockout (NIRKO) [49].

Patients with T2DM exhibit pathological changes similar to those of patients with PD, which further indicates their close correlation. Exacerbated DAergic neuronal loss and glial cell activation, along with aggravated inflammatory reactions and α-Syn accumulation in the midbrain and pancreas, were observed in diabetic mouse models [50]. Mice treated with 1-methyl-4-phenyl-1,2,3,6-tetrahydropyridine (MPTP) exhibited pancreatic and midbrain α-Syn expression [50]. Other studies found that α-Syn interacted with the Kir6.2 subunit in β cell of pancreatic islets and restricted insulin release [51].

### 3.2 GLP-1

Glucagon-like peptide 1 (GLP-1), a 30-amino acid peptide with a brief half-life, is a type of incretin. GLP-1 is mainly distributed in intestinal epithelial cells and the nucleus tractus solitarii (NTS) of the caudal brain stem. The GLP receptor (GLP-1R) is a type of G protein-coupled receptor (GPCR) that is expressed in peripheral tissues, such as the kidney, stomach, heart and CNS, including the cerebral cortex, hippocampus, and thalamus, especially in DAergic neurons of the ventral midbrain [52]. GLP-1 decreases blood glucose levels by enhancing insulin secretion and inhibiting glucagon secretion [52]. GLP-1 in the PVN decreases food intake in a presynaptic glutamate-independent manner [53]. Endogenous GLP-1R in the NTS affects food intake by mediating the satiating effects of gastric distension [54]. The activation of GLP-1R in the hindbrain also decreases food intake via the PKA/MAPK-mediated inhibition of AMP-activated protein kinase (AMPK) [55].

GLP-1 analogs contain short-acting exenatides, such as liraglutide and lixisenatide, and long-acting exenatide microspheres, such as abrutide, duraglutide, and saimaglutide. Recently, studies have found that some GLP-1 analogs play protective role in several rodent PD models. These analogs could diminish α-Syn expression and oxidative stress levels in DAergic neurons, increase TH levels in primary DAergic neurons, and improve motor symptoms in rodent models of PD [56]. Long-term liraglutide treatment in mouse models decreases DAergic neuronal loss by activating the PI3-K-AKT-CREB and cAMP-PKA-MAPK pathways or AMPK/peroxisome proliferator-activated receptor-gamma coactivator-1 αlpha (PGC-1α) signaling and ameliorates motor dysfunction [57, 58]. In a clinical study, the cognitive and memory impairments of PD patients were significantly improved after the administration of exenatide for a few weeks. The GLP-1R agonist NLY01 inhibited inflammation by reducing microglial activation in the brain, reduced DAergic neuron loss in the SN, and prolonged the lifespans of PD model mice generated by α-Syn preformed fibril (α-Syn PFF) injection into the Str [59].

### 3.3 Leptin

Leptin, composed of 167 amino acids, is secreted by adipose tissue. The brain perceives the amount of fat in the body via leptin levels to control appetite and the metabolism rate. Leptin receptors (LepRbs) are single transmembrane receptors that belong to the type I cytokine receptors of the IL-6 receptor family and are detected in the hypothalamus, hippocampus, cerebral cortex and SN [60]. Leptin suppresses the activation of neuropeptide Y (NPY)/agouti-related protein (AgRP) neurons in the hypothalamic arcuate nucleus (ARC) and enhances αlpha-melanocortin stimulating hormone (α-MSH) secretion to inhibit food intake [61].

Leptin and the nigrostriatal pathway may interact. For instance, the total DA storage in the midbrain DAergic neurons of leptin-deficient mice was shown to be reduced, leading to decreased neurotransmission capacity [62]. Furthermore, Evidente et al. found that PD patients with body weight loss had lower plasma leptin levels than those without body weight loss, and their BMIs and adipose tissue contents were also decreased [63, 64]. Another study showed that body weight gain was potentially associated with increased leptin levels and leptin resistance in PD patients after DBS in the subthalamic nucleus (STN) [65]. Interestingly, A53T mice overexpressing human α-Syn were shown to exhibit hypoleptinemia, increased hunger and lower body weight when fed both a high-calorie diet and a normal diet [66]. Orthostatic hypotension is a common nonmotor symptom in PD patients [67]. Leptin exhibits hypertensive effects by enhancing sympathetic excitability in the vasculature or at the renal level [68]. Lower leptin levels are related to orthostatic hypotension in PD patients, particularly in males [69].

Leptin can trigger signaling cascades such as PI3-K and MAPK/ERK and induce a form of positive feedback to reduce the production of apoptosis-related proteins, including caspase-9 and caspase-3, which enhances the survival of DAergic neurons [70]. Leptin treatment also rescues DAergic neurons in 6-OHDA PD mice by activating the MEK-ERK1/2 pathway, which increases the cAMP response element-binding protein (CREB) levels and thereby plays a protective role [71]. Leptin also preserves neuronal survival by increasing uncoupling protein-2 (UCP2) expression, which maintains the level of ATP and mitochondrial membrane potential in neuronal cultures treated with 1-methyl-4-phenylpyridinium (MPP^+^) [72]. Hydrogen sulfide (H_2_S) upregulates leptin expression and the Warburg effect, a phenomenon referred to as aerobic glycolysis, in the SN to prevent DAergic neuronal loss in PD mice treated with 6-OHDA [73]. Chan et al. found that the Warburg effect mediated by leptin could diminish rotenone- and MPTP-induced neuronal cell death [73].

### 3.4 Ghrelin

Ghrelin is a 28-amino acid peptide synthesized by X/A-like cells in the gastric oxyntic gland to stimulate growth hormone (GH) release and regulate energy homeostasis. The GH secretagogue receptor 1a (GHSR1a) is the major functional receptor of ghrelin and is abundantly expressed in the hippocampus, VTA, SNpc, and hypothalamus, especially in the ARC. Ghrelin mainly activates NPY/AgRP neurons and inhibits proopiomelanocortin (POMC)/cocaine- and amphetamine-regulated transcript (CART) neurons in the ARC to increase food intake [74, 75]. In recent years, a novel role of ghrelin as a neuroprotective molecule has drawn more attention.

Our laboratory reported that the total and active plasma ghrelin levels were downregulated in PD patients compared with age- and sex-matched healthy controls [76]. We also hypothesized that early DMV lesions induce ghrelin signaling disorders and GI symptoms in patients with PD [76]. The body weight gain of twenty-three PD patients treated with DBS of the STN was increased after 3 months, and their ghrelin levels were elevated [77]. Our laboratory demonstrated that ghrelin suppressed oxidative stress and apoptosis levels to salvage the loss of DAergic neurons in mice treated with MPTP [78]. Although a shortage of endogenous ghrelin or GHSR exacerbates the loss of DAergic neurons in PD mice treated with MPTP, exogenous ghrelin administration reverses the loss of DAergic neurons by activating UCP2-dependent mitochondrial mechanisms to reduce reactive oxygen species (ROS) levels [79]. Caloric restriction (CR) protects DAergic neurons by suppressing oxidative stress and preserving mitochondrial function in mice treated with MPTP [80]. Another study showed that the neuroprotective effects of CR in MPTP mice are associated with increased plasma levels of acyl ghrelin, a form that activates the GHSR to exert biological effects, which elevate AMPK levels and enhance mitochondrial function and biogenesis [81]. CR could not attenuate the SN TH neuronal loss in ghrelin KO mice [81]. The protective effect of ghrelin was also observed in PD cell models. In MES23.5 cells treated with MPP^+^, ghrelin exerts antioxidant effects, such as reversing Cyt release, decreasing malonaldehyde (MDA) levels, increasing the Bax/Bcl2 ratio and reducing caspase-3 activation [82, 83]. In MES23.5 cells treated with rotenone, ghrelin plays an antiapoptotic role in reducing cell death by reversing the mitochondrial membrane potential, restraining the activity of mitochondrial complex I, and depressing cytochrome C release from mitochondria [84].

Ghrelin also relieves nonmotor symptoms. Its agonist HM01 increases food intake and body weight in PD rats[85]. The HM01 also improves gastric emptying, augments fecal water content and output to alleviate constipation in 6-OHDA rats [85]. Idiopathic REM sleep behavior disorder (iRBD) is recognized as a symptom of the putative preclinical stage of PD. The dynamic regulation of ghrelin is impaired in PD patients with iRBD, and ghrelin excretion is disturbed, which increases the vulnerability of DAergic neurons in the SN and decreases gastric emptying. Circulating ghrelin enters the hippocampus to induce synapse formation and enhance spatial learning and memory, which might improve cognitive impairments in PD patients.

### 3.5 Nesfatin-1

Nesfatin-1 is an 82 amino acid peptide hormone encoded by nucleobindin2 (NUCB2). Nesfatin-1 is widely expressed in brain regions such as the hippocampus, NTS, DMV, and cortical areas and in peripheral tissues including gastric glands, the duodenum, and pancreatic islets, but the receptors of nesfatin-1 have not been identified. The anorexic effect of nesfatin-1 is mediated by activation of the melatonin system in the dorsal vagal complex (DVC) and oxytocin-positive neurons in the PVN [86, 87]. Nesfatin-1 also inhibits food intake through the hyperpolarization of NPY/AgRP neurons in the ARC or the decreased excitability of DAergic neurons in the DA reward pathway of the VTA [86].

Nesfatin-1 could restrain the excitability of DAergic neurons in the SN through postsynaptic inhibition [88]. According to Emir GK et al., nesfatin-1 is downregulated in PD patients, and the total oxidant status (TOS) levels in PD patients are higher than those in controls, which suggests that the oxidative process is enhanced by decreased levels of nesfatin-1 [89]. Our laboratory demonstrated that cell viability was significantly increased, the mitochondrial membrane potential was reversed, and the activation of caspase-3 in mitochondria was inhibited after the application of nesfatin-1 in MES23.5 cells treated with rotenone [90]. Subsequently, our laboratory also showed that nesfatin-1 attenuated the loss of DAergic neurons in mice treated with MPTP by activating the C-Raf-ERK1/2 signaling cascade and reduced the apoptosis of MES23.5 cells treated with MPP^+^ by improving mitochondrial dysfunction [91].

## 4. Obesity, hypercholesterolemia and PD

Many epidemiological studies have demonstrated that obesity is associated with the occurrence of PD. Increased triceps skinfold thickness in middle-aged people is related to an increased risk of PD in the future [92]. In a study of PD patients, 46.9% were overweight, and 19.2% were obese [93]. Similar phenomena occur in obese patients and PD patients, including the downward utilization of DA receptors and low level of DA in the SN and Str [94]. Studies have shown that the expression level of DA transporters and TH genes in the SN are correspondingly reduced in the posthumous brains of obese people, indicating that obesity is related to impaired DAergic function [95]. The mechanisms of obesity and PD are complicated and unclear. Case-control studies have shown that a high-fat diet (HFD), especially those high in animal fat, is a risk factor for PD. Impaired glucose tolerance and IR lesions induced by a HFD lead to high levels of MDA and endogenous neurotoxin production, which in turn damage DAergic neurons and result in dyskinesia [96]. A HFD results in increased DA consumption and weakens the anti-injury ability of DAergic neurons in the SN of mice treated with MPTP, which exacerbates the progression of PD [97]. Fat might act as a substrate in the Str of the SN to augment toxic DAergic neuronal damage [98]. It is also believed that astrocytes take up α-Syn at the axon terminal and have antioxidant neuroprotective effects. Free fatty acids in obese individuals activate astrocytes that promote the secretion of a large number of proinflammatory factors to increase neuronal damage [98].

A recent study reported that cholesterol modulates the conformational state of α-Syn and accelerates its oligomerization [99]. The treatment of neuronal culture with cholesterol results in excessive α-Syn aggregation. Another study indicated that compared with those with normal cholesterol levels, hypercholesterolemic mice showed significantly decreased numbers of TH-positive neurons, more extensive DA depletion in the Str and more extensive motor abnormalities, which might have been caused by the aggravated inhibition of mitochondrial complex-I activity and the increased level of radical hydroxyl radicals in the SN after the intraperitoneal injection of homocysteine [100]. Hypercholesterolemia aggravates DA loss in the SN and exaggerates motor symptoms in PD mice after MPTP treatment [101]. The plasma protein apolipoprotein E (Apo E) acts as a ligand for low-density lipoprotein receptors, which involves the transport of cholesterol and other lipids between cells via interaction with these receptors [102]. α-Syn and Apo E have similar structures, with both containing regions that easily bind cholesterol to affect its transfer [103]. In α-Syn transgenic mice, oxidative stress levels and the aggregation of α-Syn fibrils are accelerated by higher levels of cholesterol, oxidized cholesterol metabolites, and cholesterol synthase. It has been reported that statins, the main cholesterol-lowering drugs, decrease the risk of PD and alleviate the cognitive impairment of PD patients [104]. Statins also control microglial activation and consequently reduce neuroinflammatory mediators to protect neurons. The total and oxidized levels of α-Syn and α-Syn immunoreactive nerve fibers are significantly lower in lovastatin-treated than saline-treated α-Syn transgenic controls [105].

Diet regulation and exercise not only reduce body weight but also affect synaptic plasticity and protect against cognitive and neurological diseases. Studies have shown that long-term participation in reasonable physical exercises, such as running, boxing, swimming and dancing, has a beneficial effect on the motor and nonmotor symptoms of PD patients, which greatly improves their quality of life [106, 107]. Physical exercise also reduces the accumulation of α-Syn, inflammation and oxidative stress; increases the level of brain-derived neurotrophic factor (BDNF); and enhances the function of mitochondria to maintain cellular energy metabolism [107]. Exercise offsets the adverse impacts of a high-energy diet on synaptic plasticity and cognitive function by affecting BDNF signal transduction [108]. Recent evidence also suggests that intermittent energy restriction (IER) in PD cohorts might improve neuroplasticity and reduce the vulnerability of neurons to injury [108].

## 5. Summary

In this review, we focused on the broad crosstalk between energy metabolism and PD. PD patients show various manifestations of energy metabolism disorders in the early stages of disease, such as reduced food intake, sleep disorders, impaired thermoregulation, and weight changes. Energy metabolism-related centers are associated with these symptoms and affect the development of PD. Metabolism-related peptides influence energy metabolism and delay the development of PD through their anti-inflammatory, antioxidative, and antiapoptotic effects. People with energy metabolism-related diseases are at a higher risk of developing PD.

According to the impacts of energy metabolism on PD, potential treatment strategies could be considered from the following aspects. The changes in the energy regulation center associated with PD seem to be closely related to the propagation of α-Syn along the vagus nerve from the GI tract to the central brain area; therefore, blocking the vagus nerve or removing intestinal pathogenic factors may be beneficial for PD therapies. Considering the wide-ranging regulatory effects of metabolism-related peptides, determining the specific pathways that are most conducive for PD therapy might be necessary. Activation of metabolism-related peptide-dependent pathways might be an effective strategy to reduce neurodegeneration and ultimately inhibit the progression of PD. Physical exercise and diet control might be another strategy to reduce the occurrence of PD or delay its progression. Although the conflicting effects of energy metabolism in various PD models caution against deploying these strategies in the clinic, future preclinical studies are expected to clarify the basis of these differences. Hence, it is crucial to explore the energy metabolic mechanism in patients with PD; to understand the regulation of the energy metabolism control center, the mechanisms of metabolism-related peptides and the impacts of energy metabolism diseases in patients with PD; and to determine new therapeutic targets and formulate new treatment strategies to prevent PD progression.
